# Asexualization of the Unfit

**Published:** 1912-10

**Authors:** 


ASEXUALIZATION OF THE UNFIT.—Barr (*Pennsylvania Medical Journal*, July, 1912) has made a personal study of 4050 cases of imbecility, and found that 65.45 per cent were caused by malignant heredities; of these 25.43 per cent were due to a direct inheritance of idiocy, and 6.91 per cent to insanity. He cites the following examples of the influence of heredity in the production of the unfit and criminal members of a community. A man of thirty-eight years is the father of nineteen defective children, all of whom are living; he and his wife are mentally below par. A man has two daughters and one illegitimate grandchild, all feeble-minded. A family in seven generations number 138 individuals and records ten stillbirths, sixteen insane, seven imbeciles, three epileptics, and thirty-two with noticeable mental peculiarities; eighty are apparently normal, but are hopeless slaves of a neurotic heredity. Of fifteen imbecile girls, three were prostitutes, nine had one illegitimate child each (two being the result of incestuous intercourse with brothers), one had two illegitimate children, two epileptics had three and four idiotic children, respectively. Four feeble-minded women had forty illegitimate children. Upon these and other observations of a similar nature, Barr strongly advocates the compulsory asexualization of the unfit as the only truly effective and logical remedy. He further shows that those thus treated are themselves greatly improved, both mentally and morally.

